# An ontology-guided semantic data integration framework to support integrative data analysis of cancer survival

**DOI:** 10.1186/s12911-018-0636-4

**Published:** 2018-07-23

**Authors:** Hansi Zhang, Yi Guo, Qian Li, Thomas J. George, Elizabeth Shenkman, François Modave, Jiang Bian

**Affiliations:** 1 Department of Health Outcomes and Biomedical Informatics, College of Medicine, University of Florida, Clinical and Translational Research Building Suite 3228, 2004 Mowry Road, PO Box 100219, Gainesville, FL 32610-0219 USA; 2 Division of Hematology and Oncology, Department of Medicine, College of Medicine, University of Florida, Gainesville, Florida, USA

**Keywords:** Semantic data integration, Ontology, Semantic web, Cancer survival, Integrative data analysis

## Abstract

**Background:**

Cancer is the second leading cause of death in the United States, exceeded only by heart disease. Extant cancer survival analyses have primarily focused on individual-level factors due to limited data availability from a single data source. There is a need to integrate data from different sources to simultaneously study as much risk factors as possible. Thus, we proposed an ontology-based approach to integrate heterogeneous datasets addressing key data integration challenges.

**Methods:**

Following best practices in ontology engineering, we created the Ontology for Cancer Research Variables (OCRV) adapting existing semantic resources such as the National Cancer Institute (NCI) Thesaurus. Using the global-as-view data integration approach, we created mapping axioms to link the data elements in different sources to OCRV. Implemented upon the Ontop platform, we built a data integration pipeline to query, extract, and transform data in relational databases using semantic queries into a pooled dataset according to the downstream multi-level Integrative Data Analysis (IDA) needs.

**Results:**

Based on our use cases in the cancer survival IDA, we created tailored ontological structures in OCRV to facilitate the data integration tasks. Specifically, we created a flexible framework addressing key integration challenges: (1) using a shared, controlled vocabulary to make data understandable to both human and computers, (2) explicitly modeling the semantic relationships makes it possible to compute and reason with the data, (3) linking patients to contextual and environmental factors through geographic variables, (4) being able to document the data manipulation and integration processes clearly in the ontologies.

**Conclusions:**

Using an ontology-based data integration approach not only standardizes the definitions of data variables through a common, controlled vocabulary, but also makes the semantic relationships among variables from different sources explicit and clear to all users of the same datasets. Such an approach resolves the ambiguity in variable selection, extraction and integration processes and thus improve reproducibility of the IDA.

## Background

As the second leading cause of death, cancer is responsible for one in every four deaths in the United States [1]. In 2017, there were approximately 1.68 million new cancer cases and 600 thousand cancer deaths in the US [2] estimated by the American Cancer Society. When first diagnosed with cancer, patients ask about their prognosis, whether their cancer is relatively easy or more difficult to treat, and the likelihood of survival. There is a huge variation in survival between cancer types, stages, age groups, races/ethnicities, genders, and many other factors. For example, among some of the frequently diagnosed cancers, including lung, colorectal, breast, and prostate cancers, the 5-year overall survival rates are 18.3, 64.9, 89.7, and 98.3%, respectively [3]. These rates are much worse when the cancer is metastasized, which are 4.5, 13.9, 26.9, and 29.8% for the same types of cancers, respectively [3].

To improve cancer survival rates and prognosis, one of the first steps is to improve our understanding of contributory factors associated with cancer survival. Priori research such as the National Institute on Minority Health and Health Disparities (NIMHD) Research Framework [4] and the social-ecological model [5] recognizes that individuals are embedded within the larger social system and constrained by the physical environment they lived in. Thus, the determinants of individuals’ health span across different domains of influence (i.e., biological, behavioral, physical/built environment, sociocultural environment, and healthcare system) as well as different levels of influence (i.e., individual, interpersonal, community, and societal). Within these frameworks, cancer survival is influenced by multiple factors from multiple levels and multiple domains. At the individual level, cancer survival is influenced by not only cancer stage of diagnosis and treatment, demographics, and financial status, but also risky health behaviors such as smoking, alcohol drinking, and physical inactivity. For example, cigarette smoking is by far the most important risk factor for lung cancer; 80% of lung cancer deaths in the US were caused by smoking [2]. Beyond individual-level factors, occupational or environmental exposure to secondhand smoke, air pollution, radiation, and some organic chemicals are also significant risk factors. Further, at the contextual level, cancer survival is influenced by public policies that influence health care delivery which could impact patients’ travel distance to the treatment facility [6].

Prior epidemiologic research on cancer survival in the US, however, has primarily focused on contributory factors from the individual level due to limited data availability. Very few studies have explored contextual factors, and certainly no study has explored all possible factors together. Most of these analyses used data from a single source, such as data from a hospital (e.g., electronic health records, EHRs), a cancer registry (e.g., the Surveillance, Epidemiology, and End Results, SEER registry) or administrative claims systems (e.g., data from Centers for Medicare and Medicaid Services, CMS) [7–10]. SEER is an extremely popular data source for studying cancer survival [8–10]. However, it is important to pool heterogeneous data sets with variables beyond the individual level for integrative data analysis (IDA) that simultaneously examine as many cancer survival predictors as possible (i.e. top down approach to the model building) so that confounding effects and interactions among predictors can be fully understood. For example, the linked SEER-Medicare data give us a more complete picture of cancer patients beyond their cancer status with other clinical characteristics such as comorbidity as well as their healthcare utilization patterns [11–14]. Nonetheless, the ability to integrate risk factors of more domains and levels from other data sources such as socioeconomic status of the community from US Census data and community smoking rate from the Behavioral Risk Factor Surveillance System (BRFSS) will further advance our understanding of the determinants of cancer survival.

Nevertheless, researchers are faced with key challenges when integrating data from different sources. Data integration is a daunting task because data from different sources can be heterogeneous in syntax (e.g., file formats, access protocols), schema (e.g., data structures), and semantics (e.g., meanings or interpretations). The effort required to connect different sources is substantial due to lack of clear definitions (i.e., data semantics) of variables, measures, and constructs. Many traditional data integration techniques have been used on large scale in biomedical research [15–17], such as rule-based links (i.e., link variables from different data sources directly base on the names and definitions), data warehouses (i.e., create a new system to store a copy of the data from difference data sources, and manage the data separately from the original data systems) and ad-hoc query optimizers (i.e., re-phrasing a user’s query into multiple subqueries according to the structures of individual distributed databases) and federated middleware frameworks (i.e., link multiple applications and user interfaces to multiple data sources, act as the overarching facade across multiple applications). However, all these traditional methods did not consider the semantic knowledge, which intend to integrate information based on the meaning of the data elements. For example, how to distinguish synonyms, homonyms and related terms (e.g., different representations of the same disease using different coding standards) across different data sources. Therefore, adopting a semantic data integration approach, we propose to generate a universal conceptual representation of “information” to bridge the data heterogeneities across different sources. The “information” includes not only data elements but also their relationships, via “ontologies”. An ontology is a computational representation of a domain of knowledge based upon a controlled, standardized vocabulary for describing entities and the semantic relationships between them [18–21]. The use of ontologies can facilitate data integration in many ways, including metadata representation, automatic data verification, global conceptualization, support for high-level semantic queries, and extend beyond traditional approaches of using common data elements (CDEs) and common data models (CDMs) [22–24], especially in the biomedical domain [15, 25].

Marenco et al. developed a Query Integrator System (QIS) to address robust data integration from heterogeneous data sources in the biosciences in 2004 [26]. An ontology server was used in QIS to map data sources’ metadata to the concepts in standard vocabularies [26]. Cheung et al. developed a prototype web application called YeastHub based on a Resource Description Framework (RDF) database to support the integration of different types of yeast genome data in different sources in 2005 [27]. Lam et al. used the Web Ontology Language (OWL) to integrate two heterogeneous neuroscience databases [28] in 2005. In a follow-up study, Lam et al. designed AlzPharm that used RDF and its extension vocabulary, RDF Schema (RDFS), to facilitate both data representation and integration [29]. Smith et al. built the LinkHub system leveraging Semantic Web technologies (i.e., RDF and RDF queries) to facilitate cross-database queries and information retrieval in proteomics in 2007 [30]. In 2008, Shironoshita et al. introduced a query formulation method to execute semantic queries across multiple data services in the cancer Biomedical Informatics Grid (caBIG), named Semantic caBIG Data Integration (semCDI). Mercadé et al. developed an ontology-based application called Orymold for dynamic gene expression data annotation, integration and exploration in 2009. Based on the QIS [26], Luis et al. designed an automated approach for integrating federated databases using ontological metadata mappings in 2009 [31]. Chisham et al. created the Comparative Data Analysis Ontology (CDAO) and developed the CDAO-Store system to support data integration for phylogenetic analysis in 2011 [32]. Kama et al. built a Data Definition Ontology (DDO) using the D2RQ (i.e., a platform to provide RDF-based access over relational databases) for accessing heterogeneous clinical data sources [33]. Pang et al. developed BiobankedConnect to speed up the process of integrating comparable data from different biobanks to get a pooled data using ontological and lexical indexing in 2014 [34]. Ethier et al. designed the Clinical Data Integration Model (CDIM) based on the Basic Formal Ontology (BFO) [35] to support biomedical data integration in 2015 [36]. Mate et al. proposed an ontology-based approach to organize and describe the medical concepts of both source and target systems in order to integrate the data across different clinical and research systems [37]. Livingston et al. created an integrated knowledge base of biomedical data from multiple sources, called KaBOB, based on Open Biomedical Ontologies [38]. In 2016, Liang et al. proposed an ontology-oriented approach to represent the relations between genes, drugs, phenotypes, symptoms, and diseases from multiple information sources in aiding the analysis of psychiatric drug repurposing [39]. Similar to our approach, Kock-schoppenhauer et al. used the ontology-based data access (OBDA) model and the Ontop framework to access relational clinical databases with SPARQL queries [40]. However, most of these existing semantic data integration systems and frameworks have focused on 1) the harmonization and alignments of data elements using semantic resources; 2) creating tailored ad hoc resources for specific use cases that may not be generalizable; and 3) the integration of data from similar data sources (e.g., data from different electronic health record systems) and addressing the syntactic (i.e., data formats) and schematic (i.e., data models) heterogeneity. Very few studies have fully leveraged the reasoning ability provided by ontologically structured data. And none of the studies has used ontologies as a knowledge representation tool to document the data integration process.

This paper describes a case study of semantic data integration linking five data sets that cover both individual and contextual level factors for the purpose of assessing the association of predictors of interest with cancer survival. The main contribution of our work is that we applied an ontology-based data integration framework to integrate both individual and contextual level factors to facilitate integrative data analysis (i.e., pool heterogeneous data sets). The use of ontologies can facilitate data integration in many ways and extend beyond traditional data integration approaches. Unlike existing ontology-driven data integration methods, our study focused on encoding the different data integration scenarios explicitly using a formal and computational model with a shared vocabulary—the Ontology for Cancer Research Variables (OCRV). Our goal is not only to make the data integration process easier, but also to facilitate documentation and communication of the data integration processes between scientists. This is significant for research rigor, transparency, reproducibility as well as data reusability.

In our previous short paper [41], we prototyped an ontology-based data access approach to integrate three different datasets to support IDA of cancer survival. In this extended journal paper, we significantly expanded our ontology-based data integration framework.

- We used n-ary relations [42] in our ontology to represent relations among more than two individuals. For example, we created a ‘*ocrv:diagnosis_relation*’ class to link the ‘*ocrv:date of diagnosis*’, the ‘*ocrv:diagnosed tumor type*’ and the ‘*ncit:patient*’.
- We adopted the Time Event Ontology (TEO) [43–45] for representing events, time, and their relationships. For example, the ‘*ocrv:date of diagnosis*’ was represented as a ‘*ocrv*:*diagnosis_relation*’ instance (event) associated with an ‘teo:*timeInstance*’ (time).
- We improved the reasoning ability via using OWL restrictions [46], so that we can encode certain knowledge (i.e., constrains on properties) in the ontology. For example, in our model, current smoker is defined as patients who (1) is a current everyday/someday smoker, and (2) smoked at least 100 cigarettes in the entire life. Thus, we created restrictions for the object property ‘ocrv:*has_smoking_status*’.
- We leveraged the ontology to exam the consistency of the source data. For example, we used an individual’s ‘*ocrv:date of diagnosis*’ and ‘*ncit:birth year*’ to calculate the diagnosis age and then compared with the value directly obtained from the ‘*ncit:age at diagnosis*’ variable to check the consistency of the source data.

## Methods

Our overall goal is to facilitate the data integration needs of a theory-driven multi-level IDA of cancer survival informed by the NHMID Research Framework and the socio-ecological model with an ontology-based semantic data integration approach.

### Data integration use case: The multi-level integrative data analysis of Cancer survival

The goal of the multi-level IDA was to examine the predictive ability of cancer survival models under 3 common data integration scenarios that researchers often face in data analysis: (1) additional predictors, especially contextual factors such as county smoking rate become available through linking multiple datasets, (2) different predictors representing the same concept are available (e.g., different definitions of rurality based on either the rural-urban commuting area (RUCA) [47] codes or the National Center for Health Statistics (NCHS) [48] urban-rural classification scheme), and (3) different forms of the same predictor are available (e.g., different grouping strategy based on the raw 10-level RUCA classification to define metropolitan vs. non-metropolitan). To do so, we linked data from five different sources to evaluate discrimination performance of predictive models for breast, lung, and colorectal cancers.

### Data sources

The multi-level IDA of cancer survival was based on data of the UF Health Cancer Center Catchment Area (CCCA) from multiple sources. The UF Health CCCA is a region in north Florida that included 20 counties: Alachua, Baker, Bradford, Citrus, Clay, Columbia, Dixie, Gilchrist, Hamilton, Jefferson, Lafayette, Leon, Levy, Madison, Marion, Putnam, Sumter, Suwannee, Taylor, and Union. The individual- and contextual-level factors were extracted from 6 data sources: (1) We collected each patient’s demographic, diagnosis, treatment and survival information from the 1996–2010 Florida Cancer Data System (FCDS) [49]—a statewide cancer registry supported by the Centers for Disease Control and Prevention (CDC)—data. The FCDS followed the national data standards set forth by the American College of Surgeons, Commission on Cancer (ACoS/CoC), the North American Association of Central Registries (NAACCR) and the Surveillance, Epidemiology and End Results (SEER). (2) We obtained census tract level social vulnerability index (SVI) [50] from the Agency for Toxic Substances & Disease Registry (ATSDR). (3) We also obtained education and poverty information from the United States Census Bureau [51] (i.e., the 2000 US census). (4) We obtained county-level smoking rate, alcohol consumption rate, and health status from the Behavioral Risk Factor Surveillance System (BRFSS) [52] of the Centers for Disease Control and Prevention (CDC). Note that, each record in BRFSS is assigned with a final weight to make generalizations from the sample to the populations. BRFSS uses the raking weighting methodology, which is comprised of two parts, design weight and raking, where BRFSS final weight = Design Weight * Raking Adjustments [53]. (5) We used data from the County Health Ranking and Roadmaps [54], where we obtained county-level mental health, physical health, and primary care related information. All the raw data were in relational structures, thus, we imported and stored all of our source data in a relational database (i.e., MySQL) without any manipulations.

Adult cancer patients (18 years or older at the time of diagnosis) were identified in the FCDS data using the International Classification of Disease for Oncology 3rd edition (ICD-O-3) codes: C50.0-C50.9 for breast, C34.0-C34.3, C34.8, and C34.9 for lung, and C18.0-C18.9, and C26.0 for colorectal cancers. We obtained 50,151 unique cancer patients (18,644 breast, 21,552 lung, and 9955 colorectal). Table 1 shows a summary of the risk factors extracted from the 5 data sources. The individual-level factors were all extracted from the FCDS, reflecting individual patients’ sociodemographic and clinical characteristics. The contextual-level factors such as county level average smoking rate were linked to individual patients based on their residencies. These contextual-level factors, contributing to individuals’ cancer survivals, reflect the environmental and societal characteristics of where the individuals were embedded in. In our data analysis, these contextual-level factors were either calculated at the county level (e.g., average smoking rate from BRFSS) or the census tract level (e.g., the social vulnerability index from ATSDR), depending on the geographic resolutions of the raw data available. In addition to the risk factors, our datasets also contained variables such as survival status and cause of death (if died, and coded with the International Classification of Diseases, Ninth Revision, Clinical Modification, ICD-9-CM, codes).

**Table 1:** Summary of the risk factors extracted from different datasets

Risk factor		Data source	Reference ontology
Individual level	Race Gender Ethnicity Marital status Smoking status Insurance payer Residency: county and census tract^a^ Age at diagnosis Year of diagnosis Tumor stage Tumor type Treatment procedure	Florida Cancer Data System (FCDS)	NCIt TEO OCRV
Contextual level	Census tract SVI^b^ household composition and disability Census tract SVI minority status and language Census tract SVI housing and transportation Census tract SVI socioeconomic status	Agency for Toxic Substances & Disease Registry (ATSDR)	OCRV
	Census tract high school completion rates Census tract family poverty rates^c^	United States Census Bureau	OCRV
	Census tract rurality status^d^		OCRV NCIt
	County adult mental and physical health status^e^ County density of primary care physicians^f^	County Health Ranking & Roadmaps	OCRV
	County smoking rate County alcohol consumption rate	Behavioral Risk Surveillance System (BRFSS)	OCRV NCIt

^a^The residency of the individual at the county- and census tract-level (i.e., which county and census tract the individual lives in), which are the linkage variables used to connect the individuals with contextual-level risk factors

^b^Social Vulnerability Index (SVI) refers to the resilience of communities when confronted by external stresses on human health, such as when facing disasters or disease outbreaks

^c^The percentage of all families whose income in the past 12 months is below the poverty level

^d^The rurality status for each census tract is based on the RUCA code

^e^The average number of days a county’s adult respondents report that their mental/physical health was not good during past 30 days

^f^The ratio of the population to total primary care physicians

### Overview of a semantic data integration pipeline

**Fig. 1:**
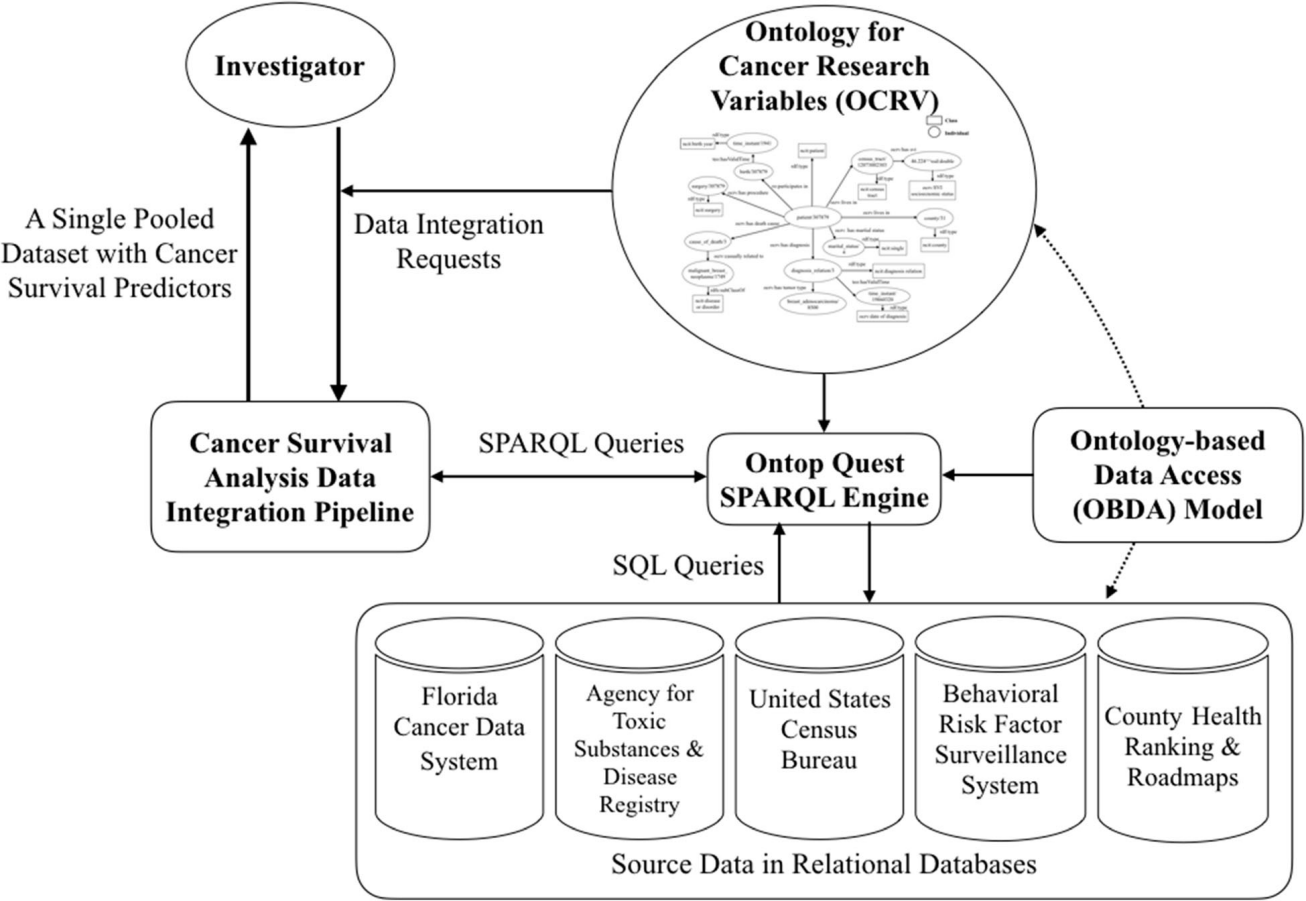
The overall process of our semantic data integration approach through an ontology-based data access framework. *The user can post data integration requests according to OCRV, then the requests were converted into SPARQL queries through the data integration pipeline. The OBDA model consists of a set of semantic mappings that specify how source data are related to the entities in the ontology. The Quest is a SPARQL query engine which uses the semantic mappings in the OBDA model to translate SPARQL queries over the ontology into SQL queries over the data sources

Our semantic data integration workflow is based on an ontology-based data access (OBDA) framework demonstrated in Fig. 1. The first step of semantic data integration is to construct synthesized, integrated descriptions (i.e., a global ontology) of the information coming from multiple sources. An ontology—the Ontology for Cancer Research Variables (OCRV)—in our case, is a metadata representation of the data elements and their semantic relationships in a both human- and machine-understandable structure. After building the ontology, an OBDA model was created—using semantic mapping axioms—to link the source data elements to the entities in OCRV. Given a global view of available data from different sources, a user can pose data (integration) requests for the selected variables (and desired representations) against our data integration pipeline, which converts the requests to a set of SPARQL queries. Based on the semantic mapping axioms defined in the OBDA model, Ontop’s Quest—a SPARQL query engine—can translate a SPARQL query over the ontology into a union of sub-queries over the data sources. The integration of the sub-query results constitutes the answer to the semantic query. The sub-queries are subject to the structure of source schemas, and often expressed in the native query languages of the sources (e.g., Structured Query Language, SQL commonly used for relational databases).

We used the Ontop platform that provides an infrastructure for querying relational databases through ontologies [55]. Within Ontop, entities (i.e., classes and properties) in the ontology are mapped to the data elements (and the relationships between the data elements) in the databases and presented as virtual Resources Description Framework (RDF) graphs. Subsequently, we can then query the virtual RDF graphs via SPQRAL queries.

### Constructing an ontology for Cancer research variables (OCRV)

#### Scope

The OCRV was built for integrating and unifying multi-level predictors of cancer survival across heterogeneous data sources. The OCRV covered a broad range of individual- and contextual-level factors, as shown in Table 1. We used the Basic Formal Ontology (BFO) [35] as the upper-level ontology, and imported the NCI Thesaurus (NCIt) and the Time Event Ontology (TEO) as the foundation for creating the OCRV.

#### Approach

Using the BFO as the overarching organization, the OCRV was developed with both top-down and bottom-up strategies to catalog relevant entities. A top-down approach was followed to identify candidate entities based on the predictors (i.e., identified through a comprehensive literature review of existing predictors related to cancer survival guided by the NIMHD Research Framework and the social-ecological model) used in the cancer survival analysis. In addition, a review of existing widely accepted ontologies (using the NCBO BioPortal [56]) was conducted to find the relevant entities that can be reused in OCRV. As shown in Table 1, many of the entities that we needed are captured in the NCIt. A bottom-up approach was then used to characterize the entities that have been identified in the top-down approach. We examined the data sources that contained these risk factors, especially the metadata (i.e., the table structures of the relational database). We inspected how these risk factors were expressed in the relational source databases as well as the relationships among the raw data elements, and determined what additional entities and relations were needed to fully represent these risk factors in OCRV.

More importantly, we considered the specific data integration use cases for supporting the multi-level IDA of cancer survival. For example, in our cancer survival analysis, we only needed to model ‘*marital status*’ as ‘*single*’ vs. ‘*married*’, while the raw data has a more fine-grained categorization (e.g., ‘*widowed*’ and ‘*divorced*’). Thus, we constructed our ontology to support the data analysis needs by declaring ‘*widowed*’ and ‘*divorced*’ as subclasses of ‘*single’*.

#### Implementation

**Fig. 2:**
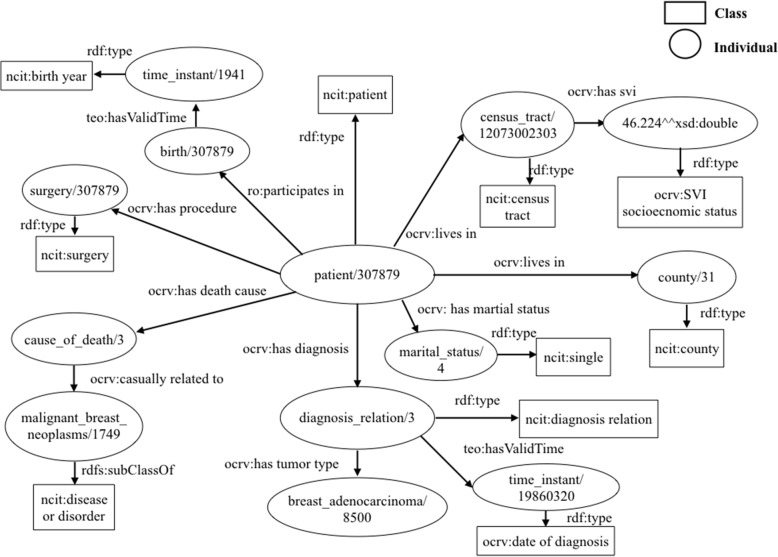
An example of data records annotated with the Ontology for Cancer Research Variables (OCRV). * Square boxes are classes, and ovals are individuals

We used Protégé 5 [57] to construct the ontology. We worked collaboratively, finalizing the list of entities based on each one’s domain expertise (e.g., oncology, cancer prevention and outcomes, and cancer population science). An example of the raw data records annotated with the OCRV is shown in Fig. 2.

### Designing the mapping axioms

In Ontop, the OBDA model consists of two parts: mapping axioms and data source declarations (i.e., to establish the database connections to the source databases using the Java Database Connectivity (JDBC) application program interface). The goal of the mapping axioms is to link the data elements in data sources to the entities in the OCRV. We constructed the mapping axioms using the Ontop Protégé plugin. In general, there were three types of mapping axioms (i.e., mappings for classes, mappings for object properties, and mappings for data properties). Each mapping axiom consisted of three fields: a mappingId, a source, and a target. The mappingId was used to uniquely identify a mapping axiom, the source is typically a SQL query to retrieve the necessary data from the data sources, and the target is an RDF triple template. Based on the OBDA model, Ontop was then able to realize the data in relational databases into virtual RDF graphs. Subsequently, we can use SPARQL queries to retrieve and manipulate the data stored in these virtual RDF graphs. Note that design of the mapping axioms as well as the ontology were driven by the data integration use cases, and subsequently, the required data query and manipulation needs. Based on the data integration needs, the SPARQL queries can be classified into four categories: (1) queries that extract variables directly linked to a patient without the need for any processing; (2) queries that need to preprocess the raw data to produce the desired results; (3) queries that are used to link a patient to contextual factors through geographic variables (e.g., county, census tract); (4) queries that generate results based on the knowledge which has been encoded in ontology. We will discuss these four types of queries in detail in the RESULTS section.

## Creating a data integration pipeline

The goal of our data integration tasks was to link predictors from different data sources to generate a single pooled dataset for cancer survival analysis. Thus, we created a data integration pipeline using the Ontop OWL Java application programming interface (API) [58] to translate user requests into SPARQL queries and to organize the query results into an analytic format. The OWL API [59] is a reference implementation for interacting with OWL ontologies. The Ontop OWL API extended the OWLReasoner interface in the OWL API to support SPARQL query answering against relational databases. As required by our data analysis models, the final results were organized into a data table (i.e., a matrix), where each row represented a patient’s cancer diagnosis record (as one patient can have multiple cancer diagnoses in the FCDS data), and each column represented a cancer risk factor.

## Results

### The ontology for Cancer research variables (OCRV)

The OCRV was constructed iteratively using the Protégé tool. We used the BFO as the upper-level ontology and imported the NCIt and the TEO as the foundation for creating the OCRV. Besides reusing terms in existing ontologies, we also created entities (i.e., classes, object properties, and datatype properties) based on the data analysis needs as shown in Table 2. Overall, we created 30 new classes, and added 23 new properties. The maximum depth of the classes is 5.

**Table 2:** The entities created for OCRV based on the data analysis needs

OCRV entity	Label
Classes	social vulnerability index • SVI household composition and disability • SVI housing and transportation • SVI minority status and languages • SVI socioeconomic status
	BRFSS current smoker • BRFSS current every day smoker • BRFSS current someday smoker • BRFSS smoker who smoked at least 100 cigarettes in the entire life
	BRFSS heavy drinker • male heavy drinker who reported having more than 14 drinks per week • female heavy drinker who reported having more than 7 drinks per week
	rural-urban commuting area codes • metropolitan area core • metropolitan area high commuting • metropolitan area low commuting • micropolitan area core • micropolitan high commuting • micropolitan low commuting • small town core • small town high commuting • small town low commuting • rural areas
	census tract high school completion rate
	census tract family poverty rate
	county adult mental health status
	county adult physical health status
	county density of primary care physicians
	single
	unknown marital status
Object properties	has death cause
	has diagnosis
	has marital status
	has procedure
	has smoking status
	has drinking status
	has tumor type
	has tumor stage
	has stage
	has race
	has biological sex
	has ethnicity
	has insurance payer
	has survival status
	has tumor stage
	has family poverty level
	has education level
	has mental health condition
	has physical health condition
	has primary care physician ratio
	has svi
	lives in
Data properties	has BRFSS final weight

**Fig. 3:**
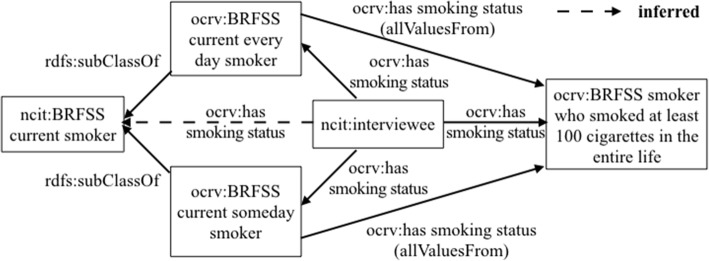
The representation of the relations among ‘*ocrv:BRFSS current every day smoker*’, ‘*ocrv:BRFSS current someday smoker*’ ‘*ocrv:BRFSS current every day smoker*’, and ‘*ocrv:BRFSS smoker who smoked at least 100 cigarettes in the entire life*’

We used *owl:Restriction* and *rdfs:subClassOf* axioms to encode the knowledge of the data integration processes in OCRV, leveraging the reasoning ability. For example, to calculate the average smoking rate for a county using BRFSS, the very first step was to find the number smokers for the county in the raw BRFSS data. BRFSS is a national telephone survey that collects state data about U.S. residents regarding their health-related risk behaviors, chronic conditions, and use of preventative services. Thus, the raw BRFSS data were participants’ survey responses, including answers to questions such as “Do you now smoke cigarettes every day, some days, or not at all?”. According to BRFSS, a smoker was defined as a person who (1) currently smokes every day or someday, and (2) has smoked at least 100 cigarettes in the entire life. The variables we used in the source data to define a smoker were: (1) SMOKDAY2 (i.e., “Do you now smoke cigarettes every day, some days, or not at all?”), and (2) SMOKE100 (i.e., “Have you smoked at least 100 cigarettes in your entire life?”). We first defined a ‘*ocrv:BRFSS current smoker*’ class, with two subclasses as: ‘*ocrv:BRFSS current every day smoker*’ and ‘*ocrv:BRFSS current someday smoker*’, which were linked to a person (i.e., ‘*ncit:interviewee’*) using the object property ‘*ocrv:has smoking status*’. We also created a class (i.e., ‘*ocrv:BRFSS smoker who smoked at least 100 cigarettes in the entire life*’) to represent people who smoked at least 100 cigarettes in their entire life. To encode the BRFSS’s definition of smoker, we applied OWL restrictions on the property ‘*ocrv:has smoking status*’ for each subclass of ‘*ocrv:BRFSS current smoker*’ as shown in Table 3 in Notation3 (N3) syntax [60], which enforces that the ‘*ocrv:BRFSS current every day smoker*’ and ‘*ocrv:BRFSS current someday smoker*’ need to have smoked at least 100 cigarettes in their entire life. Fig. 3 shows the relations among ‘*ocrv:BRFSS current every day smoker*’, ‘*ocrv:BRFSS current someday smoker*’ ‘*ocrv:BRFSS current every day smoker*’, and ‘*ocrv:BRFSS smoker who smoked at least 100 cigarettes in the entire life*’.

**Table 3:** The implementation of the OWL restrictions for ‘*ocrv:BRFSS current smoker*’

Class	Notation 3 (N3) code
BRFSS current smoker	@prefix: <http://www.semanticweb.org/ontologies/OCRV#> @prefix rdfs: <http://www.w3.org/2000/01/rdf-schema#> @prefix owl: <http://www.w3.org/2002/07/owl#> : BRFSS current smoker a owl:class rdfs:subClassOf ncit:smoking status.
BRFSS current every day smoker	@prefix: <http://www.semanticweb.org/ontologies/OCRV#> @prefix rdfs: <http://www.w3.org/2000/01/rdf-schema#> @prefix owl: <http://www.w3.org/2002/07/owl#> :BRFSS current every day smoker a owl:class rdfs:subClassOf [a owl:Restriction; owl:allValuesFrom: BRFSS smoker who smoked at least 100 cigarettes in the entire life; owl:onProperty:has smoking status].
BRFSS current someday smoke	@prefix: <http://www.semanticweb.org/ontologies/OCRV#> @prefix rdfs: <http://www.w3.org/2000/01/rdf-schema#> @prefix owl: <http://www.w3.org/2002/07/owl#> :BRFSS current someday smoker a owl:class rdfs:subClassOf [a owl:Restriction; owl:allValuesFrom: BRFSS smoker who smoked at least 100 cigarettes in the entire life; owl:onProperty:has smoking status].
BRFSS smoker who smoked at least 100 cigarettes in the entire live	@prefix: <http://www.semanticweb.org/ontologies/OCRV#> @prefix rdfs: <http://www.w3.org/2000/01/rdf-schema#> @prefix owl: <http://www.w3.org/2002/07/owl#> :BRFSS smoker who smoked at least 100 cigarettes in the entire life a owl:class rdfs:subClassOf ncit:smoking status.

**Fig. 4:**
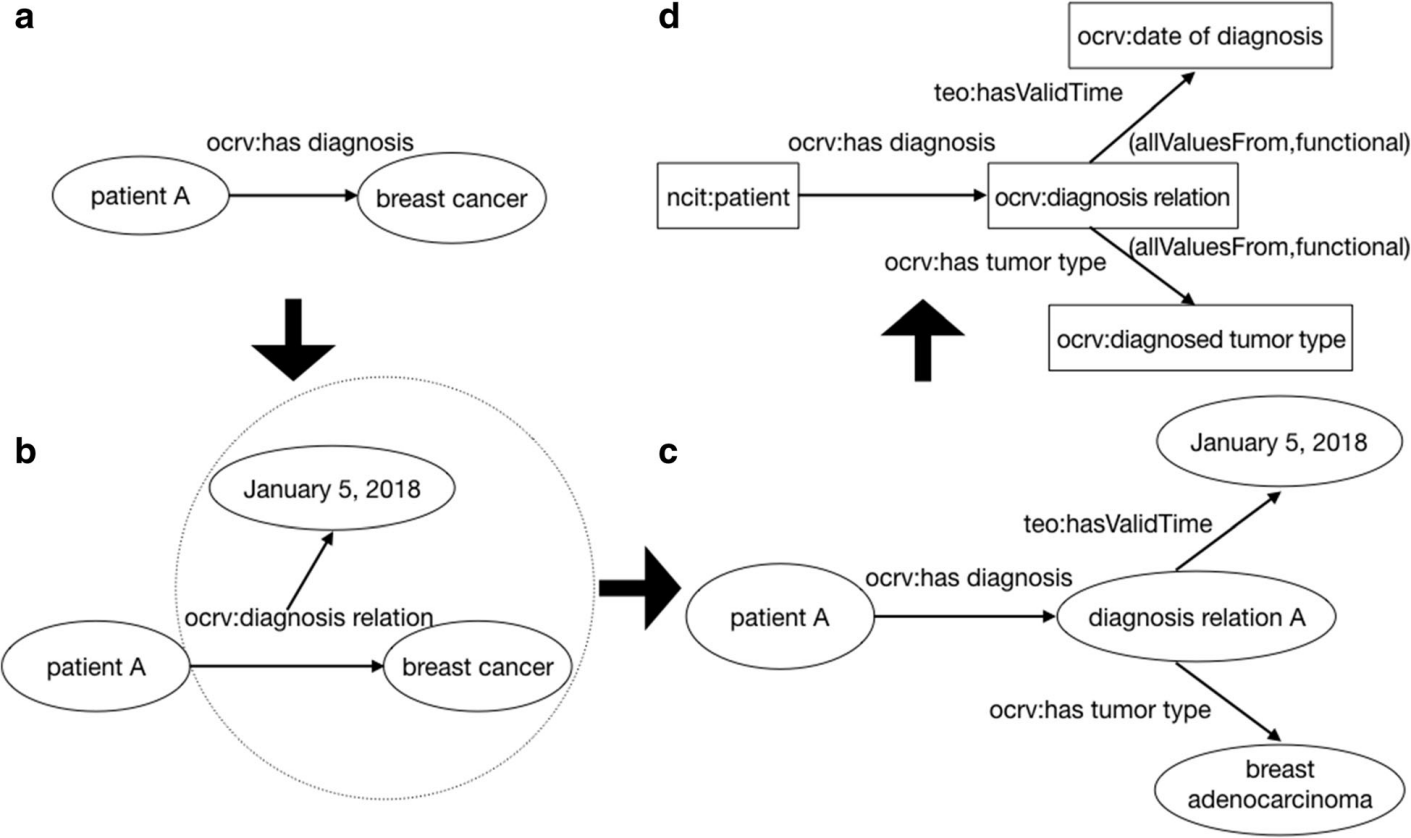
The representation of the n-ary relation pattern among patient, diagnosed tumor type, and date of diagnosis, where (**a**) illustrates how a patient A links to a breast cancer diagnosis, (**b**) is a graphical representation of the diagnosis relation contains not only the diagnosis "breast cancer" but also a timestamp for the date of diagnosis, (**c**) shows on the instance level how a patient A, her diagnosis of breast adenocarcinoma, and the diagnosis date are connected, and (**d**) shows how owl:Restriction is used to implement this n-ary relation

We also followed the best practices to define n-ary [42] relations to describe relations among more than two individuals or values. In RDF and OWL, properties are binary relations, linking two individuals or an individual and a value. For example, as shown in Fig. 4a, a patient A has been diagnosed with breast cancer. However, we also want to express that the diagnosis relation happened on a specific date (‘*date of diagnosis*’), as “*The* *patient A* *has been diagnosed with* *breast cancer* *on* *January 5, 2018.*’ This is an n-ary relation, as shown in Fig. 4b, where the object property ‘*ocrv:diagnosis relation*’ also needs to have an attribute of ‘*date of diagnosis*’, which we cannot represent. To do so, we instead created a ‘*ocrv:diagnosis relation*’ class; and a ‘ocrv*:diagnosis relation A*’ individual referring to an instance of the relation among a ‘*ncit:patient*’, a ‘*ocrv:diagnosed tumor type*’, and the ‘*ocrv:date of diagnosis*’, as shown in Fig.4.c. The individual ‘*:diagnosis relation A*’ represents a single object encapsulating both the diagnosed tumor type (‘:*breast adenocarcinoma’*, a specific instance of the tumor type) and the date of the diagnosis (‘*January 5, 2018′*). Each of the 3 statements in the original n-ary relation, who was diagnosed, what the diagnosis is, and when it was diagnosed, is then a binary relationship (i.e., ‘*ocrv:has diagnosis*’, ‘*ocrv:has tumor type*’, ‘*teo:hasValidTime*’). The class definitions for the individuals in this pattern are shown in Fig. 4d. Both properties ‘ocrv:*has tumor type*’ and ‘teo:*hasValidTime*” are functional properties, ensuring that each instance of ‘*ocrv:diagnosis relation*’ class has exactly one value for ‘*ocrv:diagnosed tumor type’* and one value for ‘*ocrv:date of diagnosis’*. The OWL restrictions on these two properties restrict the values of the properties (e.g., *owl:allValuesFrom* indicates that the values of the property are all members of the class indicated by the *owl:allValuesFrom* class). We instantiated the specific ‘*ocrv:diagnosed tumor type*’ subclasses using the ICD-O-3 codes as in the original FCDS data source (i.e., mapping axioms that linked each ICD-O-3 code to a specific tumor type as defined in the NCIt).

### The four types of SPARQL queries

#### Type 1: Queries that extract variables directly linked to a patient without the need for any processing

**Fig. 5:**
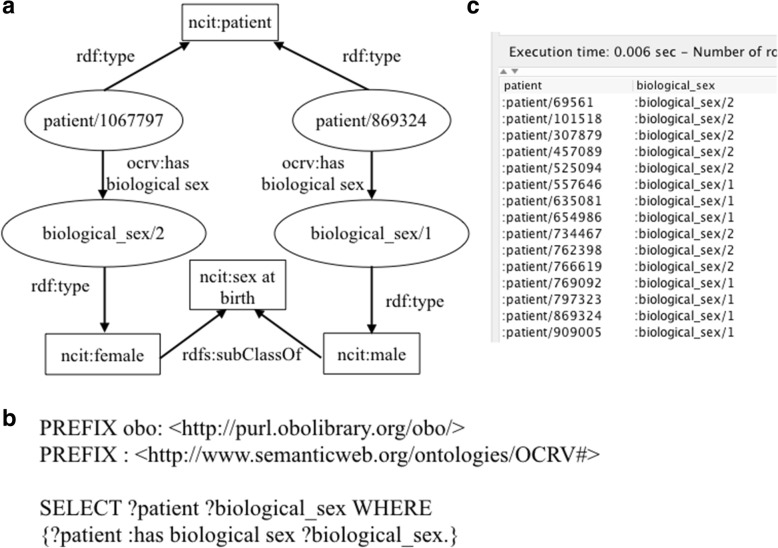
A SPARQL query that extracts the sex information of all patients, where (**a**) is a graphical representation of the relationship between a ‘*ncit:patient*’ and its ‘*ncit:sex at birth*’, (**b**) shows the SPARQL query, and (**c**) shows the query results

Many individual-level factors such as the gender of a patient from the FCDS can be extracted with a simple SPARQL query. For example, the object property ‘*ocrv:has biological sex*’ was used to link a ‘*ncit:patient’* its ‘*ncit:sex at birth*’ as shown in Fig. 5a. Based on this relation, we can use a simple SPARQL query as shown in Fig. 5b. to retrieve all patients’ sex information, where ‘*?patient*’ represents the patients and ‘*?sex*’ represents the patients’ sex at birth information. Note that, in SPARQL, query variables are prefixed with either “?” or “$”. The results of the query were shown in Fig. 5c.

#### Type 2: Queries that need to process the raw data to produce the desired results

**Fig. 6:**
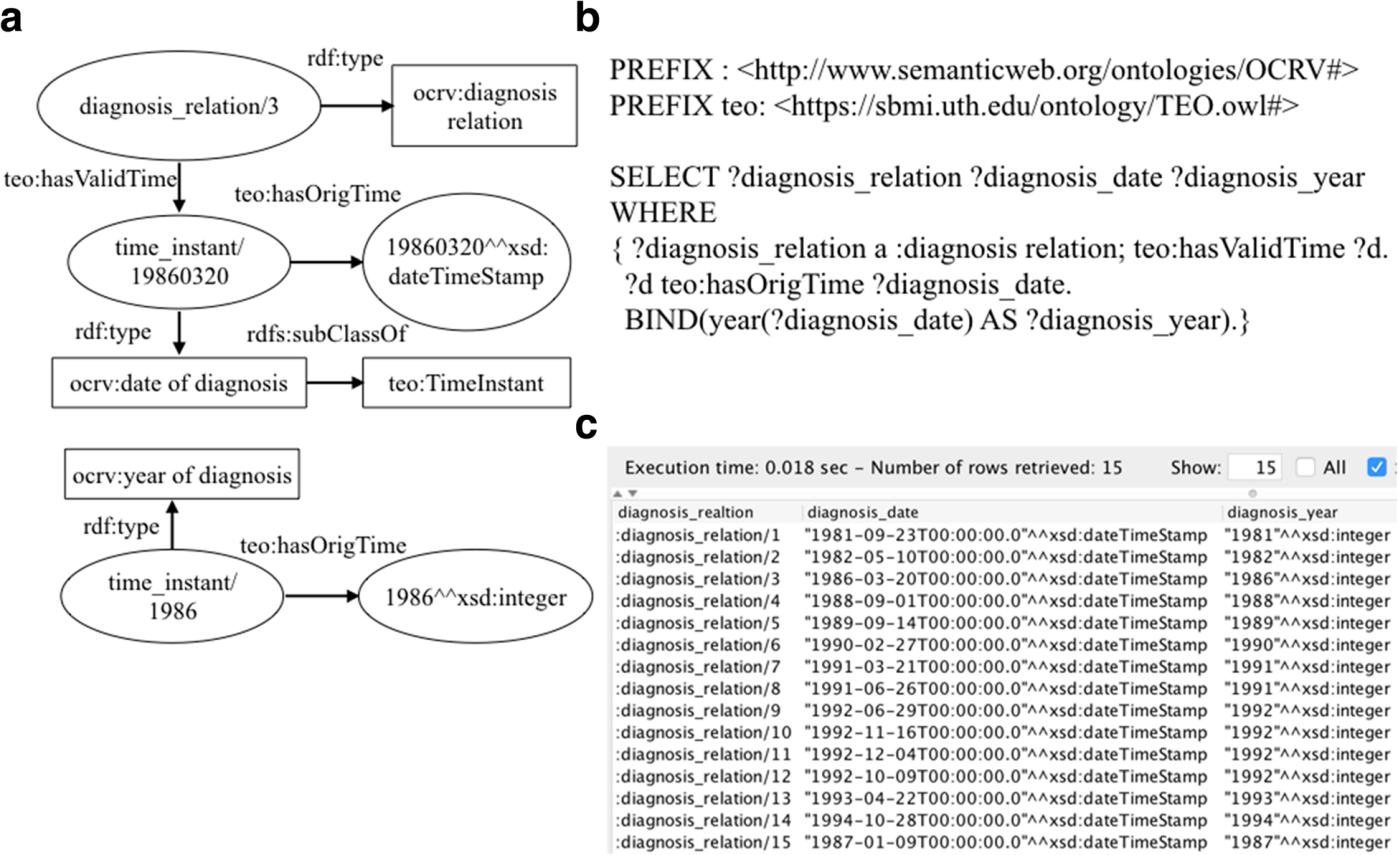
A SPARQL query that converts the date of diagnosis to the year of diagnosis, where (**a**) illustrates how a patient was linked to the date of diagnosis, (**b**) shows the SPARQL query, and (**c**) shows the query results

In data analysis, many raw variables needed to be processed to derive new variables or converted into different formats. For example, in our cancer survival analysis, we only considered the year of diagnosis, whereas the raw data in FCDS were recorded as the date of diagnosis in ‘yyyymmdd’ format. In OCRV, we used ‘*teo:hasOrigTime*’ (i.e., a data property in TEO) to link a date value to an individual of ‘*ocrv:date of diagnosis*’. Fig. 6 illustrates our process to convert the raw date of diagnosis to the desired year of diagnosis in a SPARQL query.

#### Type 3: Queries that are used to link a patient to contextual factors through geographic variables

**Fig. 7:**
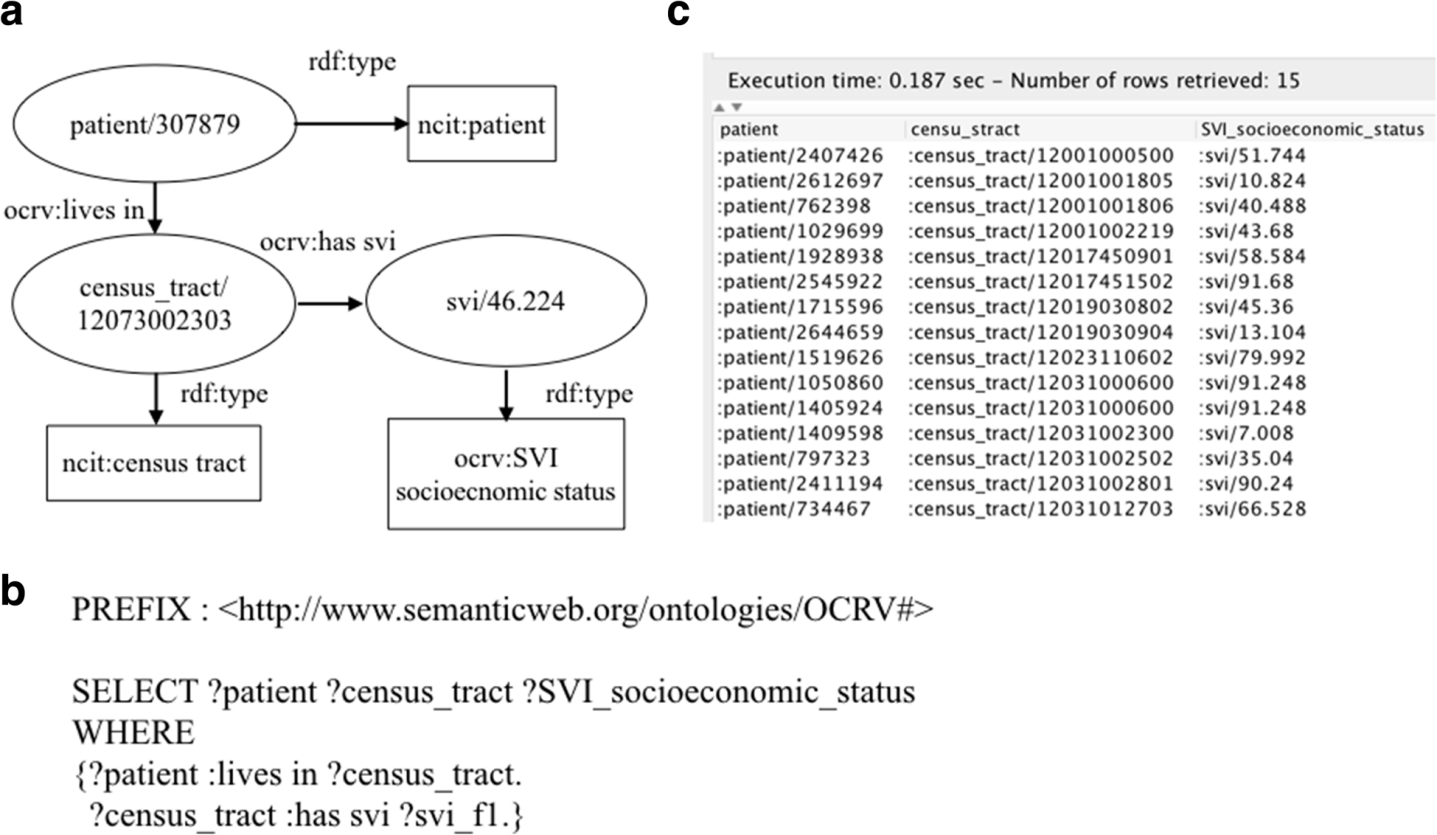
A SPARQL query that links the census tract level SVI socioeconomic status to a patient, where the *‘?census_tract*’ query variable is the common census tract code linking the individuals of ‘*ocrv:SVI socioeconomic status*’ and ‘*ocrv:patient*’. Figure (**a**) shows a graphical representation of how a patient is linked to its area-level socioeconimic status based on her residency, (**b**) is the SPARQL query, and (**c**) shows the query results

The contextual-level factors used in our cancer survival models were linked to individual patients through their residencies. In FCDS, each patient’s residency was mapped to both a census tract code and county code. We obtained the SVI (i.e., a census tract-level variable) from the ATSDR [61]. The SVI indicates the relative vulnerability of every U.S. census tract. The SVI ranks the tracts on 15 social factors, including unemployment and minority status, and further groups them into four themes: socioeconomic status, household composition & disability, minority status & language, and housing & transportation. Each tract receives a ranking for each factor and for each of the four themes, as well as an overall ranking. Based on the census tract codes, we can link a census tracts’ SVI rankings to each patient. Fig. 7 shows an example of linking the census tract level SVI socioeconomic status to a patient.

#### Type 4: Queries that generate results based on the knowledge encoded in ontology

**Fig. 8:**
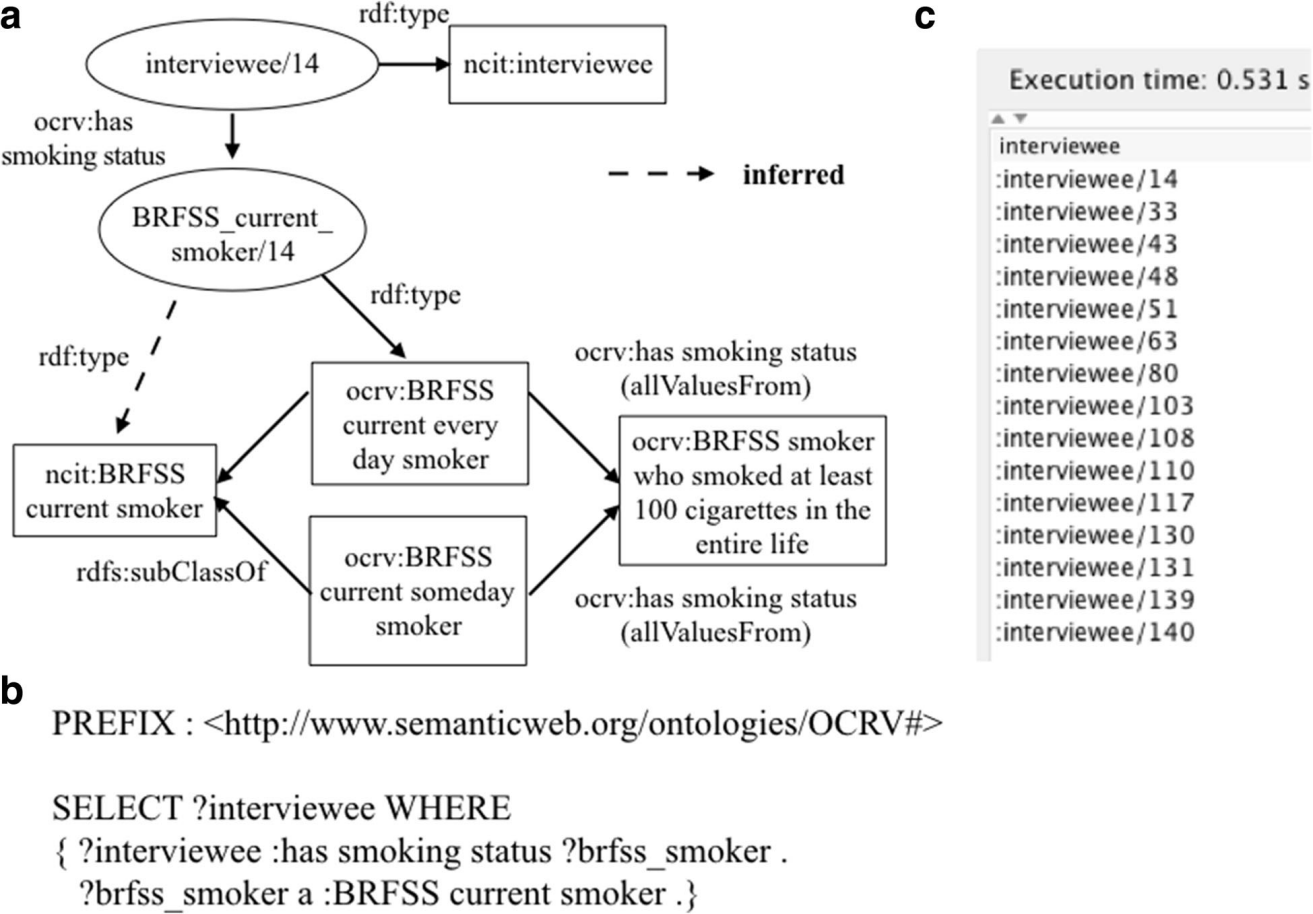
A SPARQL query that lists all current smokers in BRFSS based the OWL restrictions encoded in the ontology, where (**a**) is the graphical representation, (**b**) shows the SPARQL query, and (**c**) shows the query results

We discussed how the ‘*ocrv:BRFSS current smoker*’ was implemented in OCRV in details above. After encoded the knowledge of what is a BRFSS current smoker, a simple SPARQL query as shown in Fig. 8b can be used to retrieve all current smokers from the source BRFSS data. The reasoner can automatically resolved the subclasses of the ‘*ocrv:BRFSS current smoker’* and applied the OWL restrictions to ensure that the retrieved BRFSS current smokers meet the two conditions: (1) current every day or someday smoker, and (2) has smoked at least 100 cigarettes in the entire life.

**Fig. 9:**
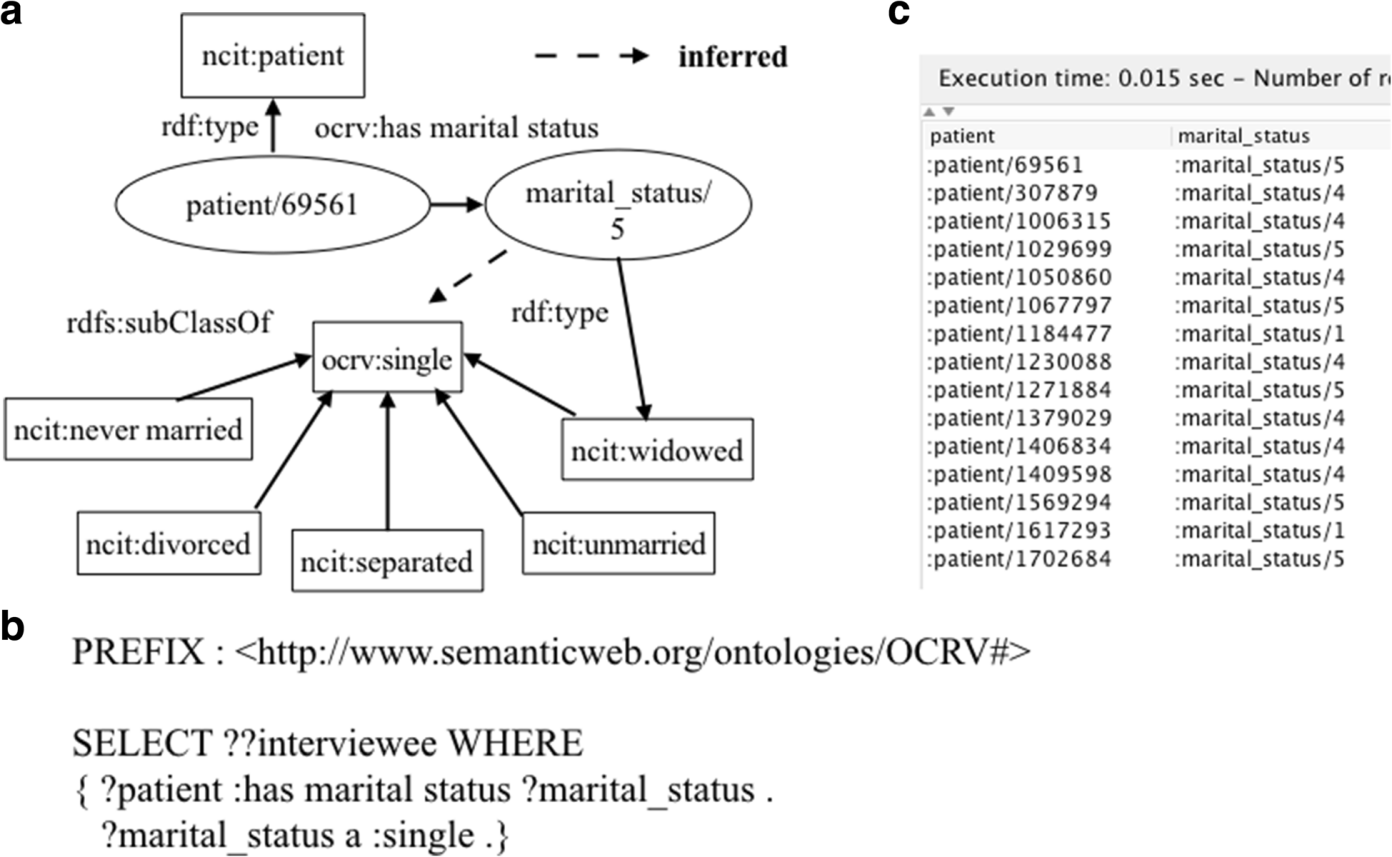
A SPARQL query that lists all patients in FCDS whose marital statuses were single leveraging the grouping logics encoded in OCRV, where (**a**) is the graphical representation, (**b**) is the SPARQL query, and (**c**) shows the query results

Also, according to our data analysis use cases, some of the raw categorical variables were regrouped into different subgroups—a common practice in building prediction models. To produce the desired grouping, we created new classes for the new groups and leveraged object properties to encode these grouping logics in OCRV. For example, the raw marital status had 7 categories (i.e., ‘never married’, ‘divorced’, ‘widowed’, ‘separated’, ‘married’, ‘unknown’ and ‘unmarried’) in FCDS. However, based on our data analysis needs, ‘never married’, ‘divorced’, ‘widowed’, ‘separated’, and ‘unmarried’ were considered equivalent to ‘single’ as shown in Fig. 9a. Thus, we created a ‘*ocrv:single*’ class in OCRV and modeled ‘*ocrv:divorced*’, ‘*ocrv:widowed*’, ‘*ocrv:separated*’, and ‘*ocrv:unmarried*’, and ‘*ocrv:never married*’ as subclasses of ‘*ocrv:single*’. Then, we can easily use a SPARQL query as shown in Fig. 9b to retrieve all patients whose marital statuses were single. The results of the query were showed in Fig. 9c.

### The semantic data integration pipeline for Cancer survival analysis

Equipped with all the necessary SPARQL queries, our last step was to build a data integration pipeline in Java with the Ontop OWL API to produce a pooled dataset for our multi-level cancer survival analysis. The process of the data integration pipeline consists of 6 main steps: (1) set up the connections among the Ontop SPARQL query engine, reasoner, OBDA model, OCRV ontology, and underlying relational data sources; (2) load and execute a query that lists all patients (i.e., patients’ unique identifiers), and use the results as the first column in the final integrated dataset; (3) load and execute the SPARQL queries corresponding to each of the risk factors that we selected, and append the results to the corresponding patients; and (4) output the final dataset in the required format (e.g., comma-separated values (CSV) format). A sample of the final integrated dataset following the process above is shown in Table 4, where each column represents a risk factor (or the outcome) and each row represents a patient record.

**Table 4:** A sample result generated using the semantic data integration pipeline for cancer survival analysis

Patient ID	Biological Sex	Marital Status	Year of Diagnosis	..	SVI Socioeconomic Status	Survival
69,561	female	single	1981	…	20.256	1
…	…	...	…	…	…	…
1,785,573	male	single	2001	…	61.632	0

## Discussion

Our experience in building an ontology-based data integration approach for linking heterogeneous datasets for multi-level cancer survival analysis has demonstrated the feasibility of using semantic data integration to resolve semantic, syntactic, and schematic heterogeneities across different data sources.

### Benefits of an ontology-based data integration model

The use of ontologies can facilitate data integration in many ways and extend beyond traditional approaches of using common data elements (CDEs) and common data models (CDMs). First, a shared, controlled vocabulary standardizes the definitions of the data elements and makes data understandable to both human (i.e., showing the preferred names for a class, and the synonyms and properties associated with it) and computers. Second, explicitly modeling the semantic relationships among data elements makes domain and data assumptions more explicit and makes it possible to compute and reason with the data. For example, the knowledge that a current smoker in BRFSS has (1) to be a current every day or someday smoker and (2) smoked at least 100 cigarettes in the entire life (as shown in Fig. 3), can be explicitly modeled in the ontology, which cannot be achieved with CDEs and CDMs. Third, ontologies enable modeling the constrains of data elements using a formal and machine-readable language, which facilitates automatic validation and assurance of data quality. Fourth, the need to shoehorn heterogeneous data into a CDM is replaced with a more flexible ontology-based metadata representation. Subsequently, integrating a new data source is simply connecting the entities among the different data sources without the need to modify the underlying database structures and data models. Such an approach avoids the error-prone, and labor-intensive extract, transform, load (ETL) processes when transforming the source data into a CDM. Last, ontology-based metadata representations make it possible to encode the different data integration scenarios explicitly using a formal and computational model with a shared vocabulary. This makes the data integration task easier and quicker, and more importantly, facilitates communication of the data integration processes between scientists. This is significant for research rigor, transparency, reproducibility as well as data reusability.

### The ability to encode data processing and data integration knowledge in the ontology

As discussed in details above, we leveraged the OWL restrictions to properly define the ‘*ocrv:BRFSS current smoker*’ in OCRV and used object properties to enable grouping of raw categorical variables into different subgroups based on the data analysis needs. These two examples illustrated the ability to reason with ontologically structured data to fulfill data processing and data integration needs. The same logics can indeed be achieved with having a human explicitly write out the specific queries in SQL. And, ultimately in our system, the SPARQL queries were translated into low-level SQL queries. However, the key difference is that in our case the computer was able to reason with the knowledge encoded in the ontology and generate the proper SQL queries rather than having a human to reason with the data and manually construct the SQL queries, which often time is error-prone and labor-intensive. However, not all data manipulation processes can be easily encoded in the ontology. Many of the data transformation procedures were achieved through built-in functions in the SPARQL queries. For example, the process of extracting the year of diagnosis from date of diagnosis had to be realized in SPARQL as shown in Fig. 5.

**Fig. 10:**
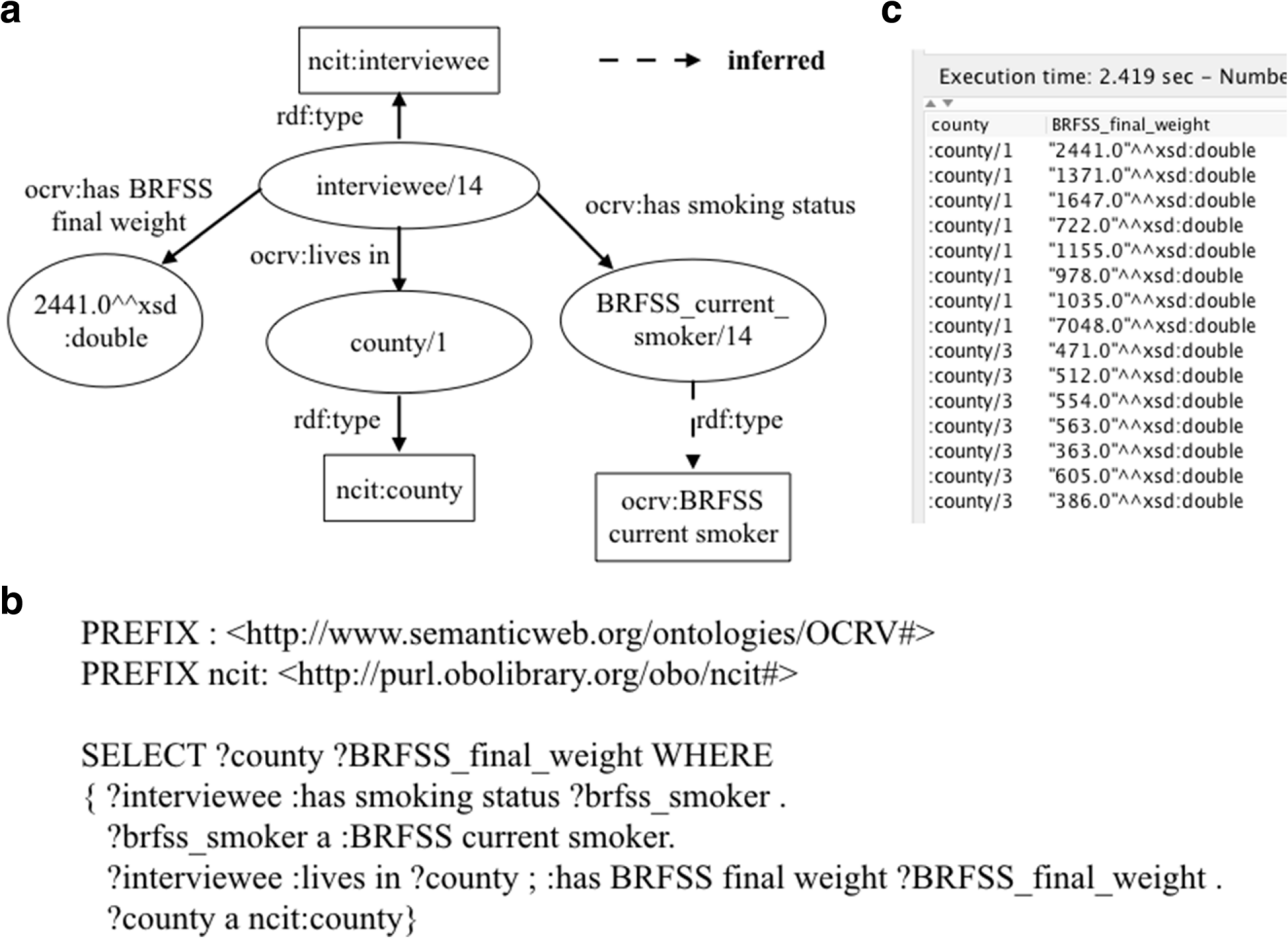
A SPARQL query that retrieves all BRFSS defined current smokers, the county codes of their residencies and BRFSS final weight, where (**a**) is the graphical representation of the relationships among these concepts, (**b**) is the SPARQL query, and (**c**) is the query results

Another example of needing computing capability in addition to the logics encoded in the ontology is to calculate the average smoking rate for each Florida county using the raw data in BRFSS. The calculation is straightforward: for each county, the average smoking rate equals to the sum of the BRFSS final weights for all current smokers divided by the sum of the final weights for all BRFSS population in the county. As shown in Fig. 3, we can identify who is BRFSS defined current smoker. A county code and a BRFSS final weight were also attached to each BRFSS respondent as shown in Fig. 10. With these information, we can easily calculate the average smoking rate for each county. Nevertheless, these computations were programed in the data integration pipeline Java code.

### Data quality and consistency checks of the source data using the ontology

**Fig. 11:**
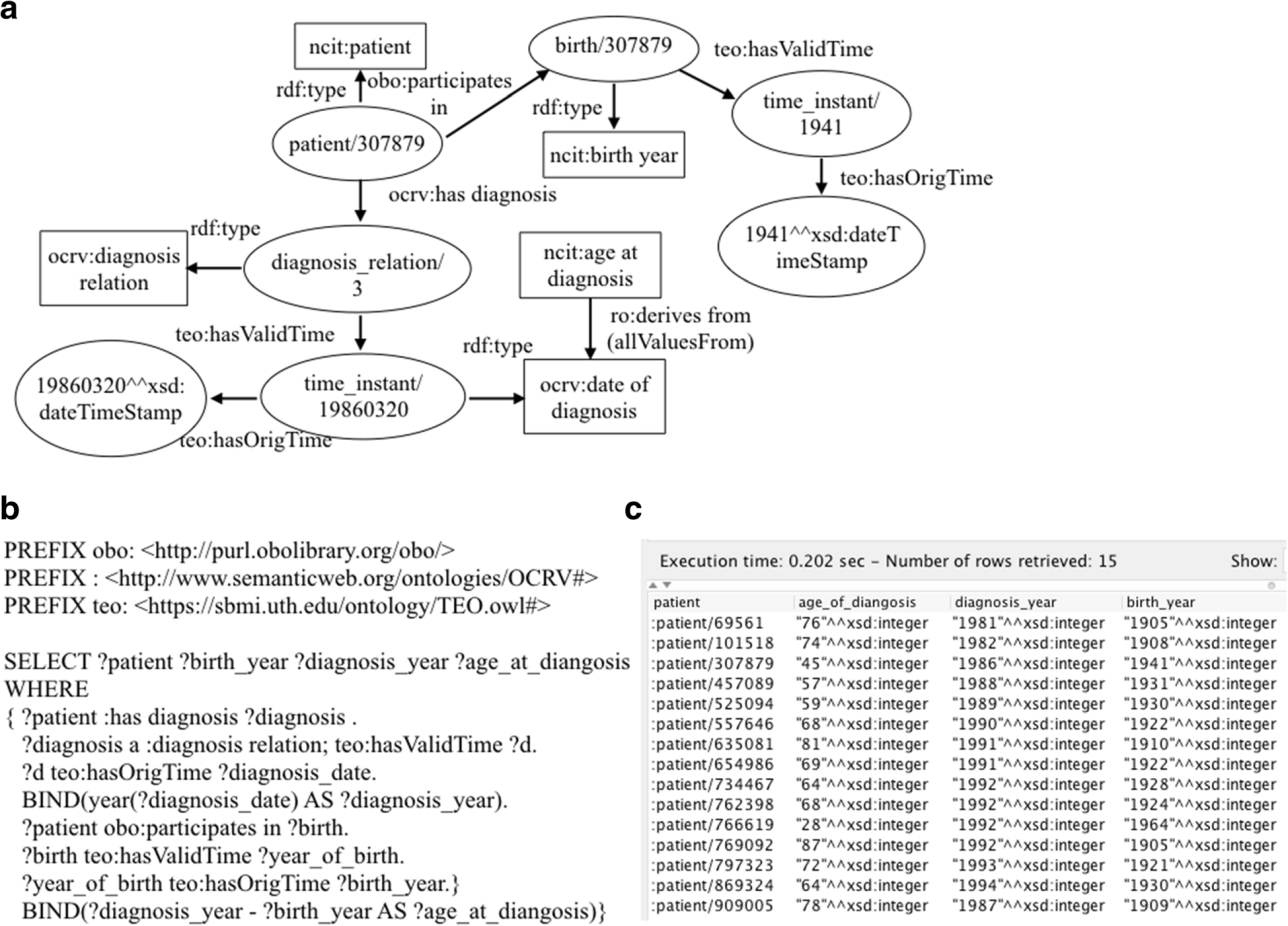
An example of using a SPARQL query to calculate the ‘*age at diagnosis*’ from the ‘*date of diagnosis*’ and ‘*birth year*’ for data consistency checks, where (**a**) shows a graphical representation, (**b**) is the SPARQL query, and (**c**) shows the query results

Another advantage of using ontology as a data model is to conduct automated data quality and consistency checks of the source data, because the dependencies among and constraints of the data elements are explicitly modeled in the ontology. For example, FCDS has two related variables: ‘*age at diagnosis*’ and ‘*date of diagnosis*’. As shown in Fig. 11, we explicitly modeled the relationship and constraints between these two entities, as the ‘*ncit:age at diagnosis*’ can be calculated from the ‘*ocrv:date of diagnosis*’ and ‘*ncit:birth year*’. We can then compare the raw ‘*age of diagnosis*’ and the computed age of diagnosis to exam data consistency. Further, we also used OWL restrictions to enforce the required formats and ranges of the data elements. Equipped with automated data consistency checks, data analysts shall follow best practices in dealing with data quality issues [62].

### Limitations and future work

The current OCRV stemmed from our particular IDA for cancer survival use case does not cover all possible modeling needs in cancer research. More use cases need to be considered to expand our ontology. In addition, we instantiated classes ‘*ncit:disease or disorde*r’ and ‘*ocrv:diagnosed tumor type*’ ICD-9-CM code and the International Classification of Diseases for Oncology 3rd Edition(ICD-O-3), respectively, through Ontop’s mapping axioms. Currently, the knowledge of linking a specific disease to the corresponding ICD-9-CM/ICD-O-3 codes is manually expressed in the SQL queries when creating the mapping axioms. In the future work, we will import the disease ontology, where the corresponding ICD codes have already been encoded in the classes as annotation properties. Nevertheless, we will need to extract and convert these annotation properties to data properties (i.e., create individual mapping axioms linking the individuals of diseases to individuals of ICD codes). Consequently, the reasoner can leverage these mapping axioms to infer the knowledge of how to connect a set of ICD codes to corresponding diseases automatically.

## Conclusions

We have presented an ontology-based semantic data integration approach for multi-level integrative data analysis of cancer survival. Our approach solves key data integration challenges: (1) not only provide clear definitions of the data elements using standardized common vocabulary, but also explicitly expressed the relationships among the variables, (2) be able to clearly document data processing and integration procedures, (3) leverage ontologically structured data to infer and derive the required data elements and formats automatically, (4) provide a convenient mechanism for documenting data quality and consistency checks, and most importantly (5) allows scientists to clearly document and communicate their data manipulation processes, which is significant for research rigor, transparency, reproducibility as well as data reusability.

## Abbreviations

ACoS/CoC: American College of Surgeons, Commission on Cancer
API: Application Programming Interface
ATSDR: Agency for Toxic Substances & Disease Registry
BRFSS: Behavioral Risk Factor Surveillance System
caBIG: cancer Biomedical Informatics Grid
CCCA: Cancer Center Catchment Area
CDC: Centers for Disease Control and Prevention
CDE: Common Data Element
CDIM: Clinical Data Integration Model
CMD: Common Data Model
CMS: Centers for Medicare and Medicaid Services
CSV: Comma-Separated Values
DDO: Data Definition Ontology
EHR: Electronic Health Record
ETL: Extract, Transform, Load
FCDS: Florida Cancer Data System
ICD-9-CM: International Classification of Diseases, Ninth Revision, Clinical Modification
ICD-O-3: International Classification of Diseases for Oncology 3rd Edition
IDA: Integrative Data Analysis
JDBC: Java Database Connectivity
N3: Notation 3
NAACCR: North American Association of Central Registries
NCHS: National Center for Health Statistics
NCI: National Cancer Institute
NIMHD: National Institute on Minority Health and Health Disparities
OBDA: Ontology-Based Data Access
OCRV: Ontology for Cancer Research Variables
OWL: Web Ontology Language
QIS: Query Integrator System
RDF: Resource Description Framework
RDFS: Resource Description Framework Schema
RUCA: Rural-Urban Commuting Area
SEER: Surveillance, Epidemiology, and End Results
semCDI: Semantic caBIG Data Integration
SVI: Social Vulnerability Index
TEO: Time Event Ontology
